# Innovative Application of Chicken Eggshell Calcium to Improve the Functional Value of Gingerbread

**DOI:** 10.3390/ijerph19074195

**Published:** 2022-04-01

**Authors:** Marcellus Arnold, Yolanda Victoria Rajagukguk, Andrzej Sidor, Bartosz Kulczyński, Anna Brzozowska, Joanna Suliburska, Natalia Wawrzyniak, Anna Gramza-Michałowska

**Affiliations:** 1 Department of Gastronomy Science and Functional Foods, Faculty of Food Science and Nutrition, Poznań University of Life Sciences, Wojska Polskiego 31, 60624 Poznań, Poland; marcellus.arnold@up.poznan.pl (M.A.); yolanda.rajagukguk@up.poznan.pl (Y.V.R.); andrzej.sidor@up.poznan.pl (A.S.); bartosz.kulczynski@up.poznan.pl (B.K.); anna.brzozowska@up.poznan.pl (A.B.); natalia.wawrzyniak@up.poznan.pl (N.W.); 2 Department of Human Nutrition and Dietetics, Faculty of Food Science and Nutrition, Poznań University of Life Sciences, Wojska Polskiego 31, 60624 Poznań, Poland; joanna.suliburska@up.poznan.pl

**Keywords:** antioxidant, chicken eggshell, green tea, functional food, osteoporosis, radicals

## Abstract

Food waste, such as eggshell, can be an environmental problem if it is not properly managed. One of the ways to solve this is by using the eggshell as the cheap calcium source in food products. Polish gingerbread fortified with chicken eggshell powder (ESP) calcium was developed to solve the eggshell waste problem and to reduce the risk of osteoporosis. This study focused on the effect of ESP addition on basic composition, sensory evaluation, and antioxidative activity of gingerbread. Two samples of gingerbread without and with 3% (*w*/*w* of wheat flour) ESP, with controlled green tea powder (4% *w*/*w* of white chocolate) were analyzed. Results of the research showed that the addition of 3% ESP significantly increased the ash and calcium content (*p* < 0.05) without changing the appearance, aroma, texture, taste profiles, and the hedonic score of gingerbread. The gingerbread samples were then stored for 2 months and were analyzed every month. The hedonic evaluation of the aroma of both gingerbread samples decreased significantly (*p* < 0.05) during storage. During 2 months of storage, the antioxidative activity of gingerbread fortified with 3% ESP was not significantly different compared to the control (*p* > 0.05), particularly in ABTS and ORAC_FL_ assay. The ABTS, DPPH, and ORAC_FL_ assays showed decreasing antioxidative activity during storage, which was also in accordance with decreasing total phenolic content of both gingerbread samples. In PCL assay, the lipid-soluble antioxidant activity in gingerbread with 3% ESP was significantly higher during 2 months of storage, compared to the control (*p* < 0.05). The developed product might be a potential alternative to improve the calcium (26% daily value (DV) recommendation per 100 g) and antioxidant intake in order to prevent calcium deficiency. Gingerbread enriched with an organic source of calcium may become an innovative product to reduce the risk of developing osteoporosis in the elderly population, having potential health and economic significance, given the incidence of osteoporosis and the costs of treating this disease.

## 1. Introduction

One of the major health problems in the world is calcium deficiency, which then might lead to osteoporosis. About 200 million people in the world suffer from osteoporosis [1]. Recent publications have studied the effective ways to improve calcium intake, one of which is through functional food [2,3]. Chicken eggshell, which contains around 38% calcium, has been studied to be a natural source of calcium (as calcium carbonate) that can be added to various food products, such as bread [4], biscuit [5], yogurt [6], Polish bread spread (“ser smażony”) [7], etc. The calcium absorption from chicken eggshells was reported by several studies. In a rat study, the calcium absorption of chicken eggshells was 45.59%, not significantly different from the absorption of the supplement CaCO_3_ [4]. The calcium absorption of chicken eggshells was even better than that of CaCO_3_ in a soy protein-based diet, while no difference between them was found in casein-based diets of piglets [8]. An in vitro study also reported that the bioavailability of chicken eggshell calcium could be increased by the addition of citric acid, ascorbic acid, and hesperidin [9]. Not only CaCO_3_, which constituted 94–96% of the composition of eggshell, but other substances were also found in eggshells: water, organic matter (4%), such as protein and lipid, and the other inorganic matter, such as magnesium carbonate (1%) and calcium phosphate (1%) [10,11].

Recently, the European Commission has been promoting the utilization of products’ values, resources, and materials as much as possible to support zero waste practices, which are then beneficial for the economy and environment [12]. The waste problem from animal by-products, including eggshells, has been addressed as this may negatively affect not only health problems, but also environmental problems, such as waste disposal into landfills that causes pollution by production of foul odors and promotes the spread of pathogens. From 2009 to 2019, the worldwide production of chicken eggs increased more than 30% [13], which then contributed to the waste problem from the eggshell. Therefore, it is very important to utilize the value of chicken eggshell instead of leaving it as only a waste. The usage of eggshell waste to support sustainability has been classified as raw materials (food additives, soil amendment, purified CaCO_3_, cosmetics, and biomaterial composites) and operating supply (catalyst and sorbent) [14]. As an adsorbent, the eggshell powder was reported to be a good alternative for neutralized soybean oil bleaching [15]. Among the recent utilizations of eggshell waste in industry, eggshell waste application in human diet, especially in various food products, has been developing as it will effectively increase the calcium intake for calcium-deficient people in the world, including low-income countries [16]. Therefore, in this study, the application of chicken eggshells as a food additive was the main focus.

Antioxidants (e.g., vitamin C and E, polyphenols, and lycopene) contribute as the anti-radicals to prevent oxidative stress, thus could reduce the risk of osteoporosis [17,18]. Green tea (*Camellia sinensis*) was reported to exhibit strong antioxidative activity due to its polyphenols, flavonoids including flavanols (catechins, procyanidins), flavonols (quercetin, rutin, kaempferol), and phenolic acids (gallic acid, caffeic acid) [19]. The main catechins found in the tea are (-)-epigallocatechin-3-gallate (EGCG), (-)-epigallocatechin (EGC), (-)-epicatechin-3-gallate (ECG), and (-)-epicatechin (EC) [20]. Thanks to their antioxidative and estrogen-like effect, the polyphenols may take part in increasing osteoblasts differentiation, and on the other side, also inhibiting the osteoclasts differentiation and activation [21]. Several studies on the effect of tea on osteoporosis were reported. Based on 17 journal articles, a meta-analysis study concluded that tea consumption can reduce the risk of osteoporosis [22]. Tea drinking habit was reported to benefit bone health in middle-aged and elderly men [23], men and women [24], elderly Japanese women [25], Chinese, and Iranian women (but not associated in men) [21,26]. Furthermore, the antioxidative activity from green tea polyphenols had a role in reducing the aging-induced bone loss in middle-aged female rats [27]. In a female ovariectomized rat study, Lin et al. [28] indicated that the green tea’s EGCG role in the upregulation of BMP-2 (bone morphogenetic protein-2) may be partially related to the acceleration of fracture healing. Besides antioxidative effects, tea also gives antiviral effects, anticarcinogenic effects, anti-inflammatory effects, cardioprotective effects, and prevention of neurodegenerative effects [29]. Furthermore, tea also improves the pleasant aroma and taste of food products [30]. These beneficial properties of green tea have become the reasons for the green tea-based functional foods development as opposed to the usage of chemical additives. However, the combination of green tea and calcium to improve the functional value of food products has been scarcely reported.

In this research, Polish gingerbread (in Polish: pierniki) incorporated with chicken eggshell and green tea powder was developed to increase the calcium and antioxidant intake, as well as to solve the eggshell waste problem. Polish gingerbread is a popular bread that contains flour, honey, and various spices including cinnamon, cloves, coriander fruit, allspice, and nutmeg. Gingerbread is not only just food, but also part of the tradition and history in Poland, Europe, and world, especially during Christmas tradition [31,32]. The history of food is deeply related to economy, policy, local traditions, and innovative technology [33]. In 2019, Poland was reported to be the third-largest producer (17% of total production in the European Union or EU) of gingerbread in the EU, after Germany (45%) and the Netherlands (22%) [34]. Unfortunately, the gingerbread tradition in the EU is diminishing as the statistics showed that from 2009 to 2019, gingerbread production in the EU decreased 17% by weight [34]. The developed gingerbread in this study was not only about improving the functional values of the gingerbread itself but also raising consumers’ awareness and interest in gingerbread tradition from the health point of view. Gingerbread enriched with an organic source of calcium may become an innovative product to reduce the risk of developing osteoporosis in the elderly population. The results of this research will have health and economic significance, given the incidence of osteoporosis and the costs of treating this disease.

The idea to use gingerbread as the food matrix in this study was in accordance with previous studies. The application of chicken eggshell powder in bread products is recommended as it minimally changes the texture and taste of the resulting product [4]. Bread products are also preferred by the elderly, who are at a greater risk of osteoporosis, from different regions in the world [18]. A study reported that wheat bread supplemented with the antioxidant from onion brought a significant improvement in antioxidant enzyme activities of diabetic rats [35]. Pastoriza et al. [36] reported the positive effect on the antioxidant defense in the liver of rats after a long-term Maillard reaction products-containing bread crust diet, manifested in increased antioxidant enzyme activities and glutathione. Additionally, although the formation of acrylamide, a probable carcinogen, has been related to bakery products [37], the use of sodium bicarbonate in comparison to ammonium bicarbonate as a baking agent, has been reported to successfully reduce more than 60% acrylamide formation in gingerbread production [38].

In this study, it was decided to apply the eggshell in gingerbread since it is a popular snack and willingly eaten by children and the elderly, hence becoming a source of essential calcium for healthy bone growth. Moreover, the presence of vitamin D, inulin, as well as other indigestible oligosaccharides, magnesium, or short-chain fatty acids, is beneficial for calcium absorption in the body [18,39,40]. The novelty of this article was the combined application of calcium from eggshell powder and antioxidant from green tea in gingerbread as a new alternative to increase calcium intake and reduce the risk of osteoporosis. By using the eggshell powder, this study also tried to solve the food waste problem, which becomes a major challenge in environmental management. The aims of this study were to evaluate the basic composition, calcium content, antioxidative activity, and sensory properties of gingerbread fortified with calcium from chicken eggshell powder, in order to find an alternative food product dedicated to reducing the risk of osteoporosis.

## 2. Material and Methods

### 2.1. Preparation of Eggshell Powder and Gingerbread

The eggshell powder was prepared according to Ray et al. [41] with slight modification. The collected chicken eggshells were washed and scrubbed using a sponge and running water. The clean eggshells were then boiled in water for 30 min. The drying and sterilizing process was conducted using a hot air oven (Nabertherm, Lilienthal, Germany) at 134 °C for 15 min. A grinder (Bosch, Germany) was used to grind the sterilized eggshells. The eggshell powder (size ≤ 0.315 mm) was collected after the sieving process (Haver & Boecker, Germany, 0.315 mm opening).

The ingredients used in the preparation of gingerbread samples were: UHT milk 3.2% fat (Polmlek Raciąż sp. z o.o., Poland), unrefined cane sugar (Pfeifer & Langen sp. z o.o., Poland), dry yeast (Bakalland S.A., Poland), white flour type 650 (Przedsiębiorstwo Zbożowo-Młynarskie PZZ w Soisławiu S.A., Poland), oat bran (Sante A. Kowalski sp.j., Poland), salt (CIECH Soda Polska S.A., Poland), honey (Huzar sp. z o.o., Poland), egg (Fermy Drobiu Mizgier Tomasz Mizgier, Poland), baking soda (FoodCare sp. z o.o., Poland), gingerbread spice mix (containing sugar, cinnamon 15%, cloves, fat-reduced cocoa, coriander fruit, allspice, nutmeg) (Prymat sp. z o.o., Poland), butter 82% fat (SM Mlekovita, Poland), white chocolate (Millano sp. z o.o., Poland), green tea powder (eherbata.pl sp. z o.o., Poland), and eggshell powder.

**Figure 1:**
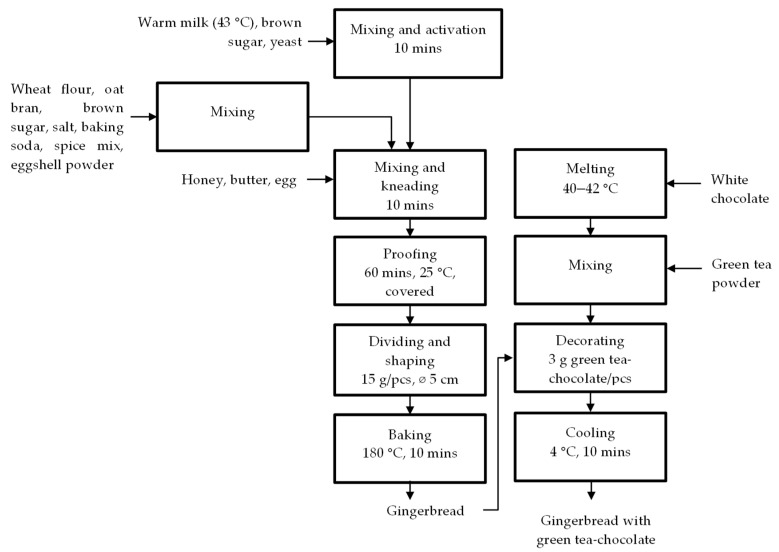
Process flow diagram of gingerbread production.

**Figure 2:**
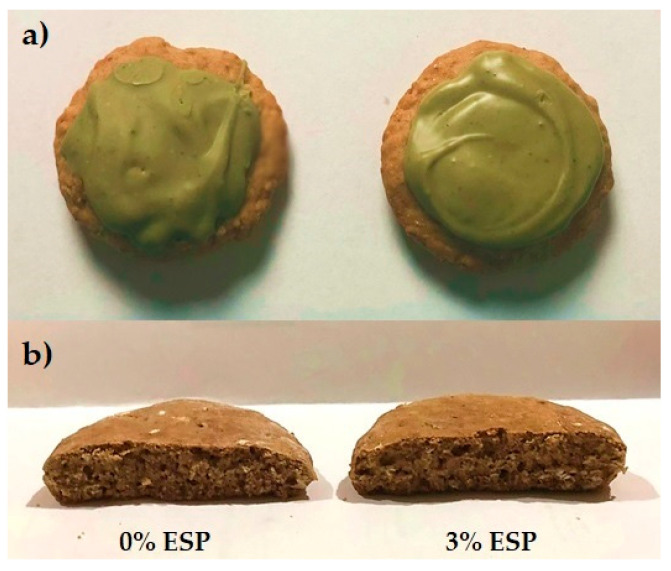
Gingerbread samples with (**a**) and without green tea chocolate topping (**b**); ESP: eggshell powder.

**Figure 3:**
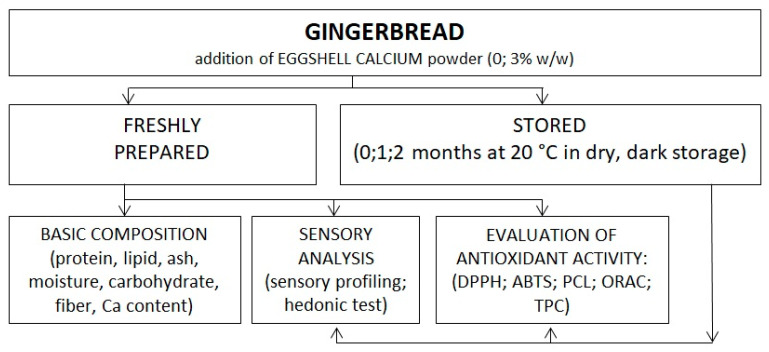
The research scheme.

The formulation and production process of gingerbread is shown in Table 1 and Figure 1, respectively. The concentration of eggshell powder (3% *w*/*w* of flour) and green tea powder was determined after considering prior research [41,42]. The previous study reported that the addition of eggshell powder from 3 to 6% *w*/*w* of flour to the cake was still acceptable in the sensory analysis [41]. However, from the preliminary evaluation of the present study (data not shown), the addition of eggshell powder more than 3% *w*/*w* of flour resulted in a more noticeable undesired typical eggshell aroma. The used concentration of green tea powder was 4% (*w*/*w*) of white chocolate topping in both control and treatment samples. The addition of green tea powder higher than 4% resulted in unacceptable taste due to high astringency and grassy aftertaste (data not shown). The resulting gingerbread with green tea-chocolate topping (Figure 2) was analyzed further (Figure 3).

### 2.2. Basic Composition Analysis

The basic composition of gingerbread samples included lipid, protein, ash, moisture, carbohydrate, fiber, and calcium content. Soxhlet method [43] using Soxtec-HT6 System (Foss Tecator, Hoganas, Sweden) was conducted in lipid extraction. The Kjeldahl method with the use of Kjeltec-2200 System (Foss Tecator, Hoganas, Sweden) following AOAC [44] was conducted to determine the protein content. The ash content was determined after complete incineration of the sample at 530 °C for 8 h, using a muffle furnace, according to AOAC [45]. For moisture content determination, the samples were dried in the oven at 105 °C (Nabertherm, Lilienthalm Germany). The weight of the sample was recorded several times and placed back in the oven until the weight was stable. The carbohydrate content was obtained after calculation of 100%, subtracted by the total sum of lipid, protein, moisture, and ash content. The energy value was calculated using the following conversion factors: carbohydrate—4 kcal/g, protein—4 kcal/g, and lipid—9 kcal/g [46]. The determination of fiber content was measured as total dietary fiber (TDF), insoluble dietary fiber (IDF), and soluble dietary fiber (SDF) following the enzymatic-gravimetric Asp method [47]. Other fiber fractions, including neutral detergent fiber (NDF), acid detergent fiber (ADF), lignin (ADL), and cellulose (ADC), were determined following Van Soest assay (Fibertec, Foss Tecator, Sweden) [19].

Prior to the determination of calcium content, the complete mineralization of 1 g of samples (powdered form) at 450 °C in a muffle furnace was conducted. It was then dissolved in 1 mol/L nitric acid (Suprapure, Merck). Analysis of the sample was conducted in triplicate. The samples were diluted with 0.5% LaCl_3_. Atomic absorption spectrometry (Hitachi Z2000) using an air-acetylene flame [48] was conducted to determine the calcium content of gingerbread samples.

### 2.3. Total Phenolic Content and Antioxidative Activity Analysis

For the evaluation of total phenolic content and antioxidative activity of gingerbread samples during storage, the samples were stored in a thermostat (20 °C) in a dark condition for 2 months. This period of storage was decided based on the approximate time that the consumer could store the product on a shelf without quality loss.

Total phenolic content (TPC) was determined following the method in Shahidi and Naczk [49], based on the reduction of Folin–Ciocalteu reagent complexes, measured at λ = 725 nm, expressed as mg of gallic acid equivalent (GAE) per 100 g of product. The standard curve of y = 4.4721x − 0.024 (R^2^ = 0.9968) was used to calculate the TPC. The preparation of antioxidant extracts was done according to Jan et al. [50], with modification. Prior to antioxidant activity analysis, the samples (3.5 ± 0.1 g) were lyophilized, ground, and extracted using 80% methanol (100 mL) for 2 h at 20 °C. 

The antioxidative activity of gingerbread samples was determined using ABTS, DPPH, ORAC_FL_, and PCL assays. The ABTS radical cation scavenging activity of gingerbread was measured according to Re et al. [51], based on spectrophotometric measurement (λ = 734 nm) of the antioxidant ability to scavenge blue-green colored ABTS (2,2′-azinobis(3-ethylbenzothiazoline-6-sulfonic acid) formed from ABTS by oxidation with potassium persulfate. In the presence of antioxidants, the ABTS cation radical intensity decreases [52]. The standard curve of y = 182.14x − 2.5547 (R^2^ = 0.9986) was used to evaluate the ABTS radical cation scavenging percentage. The result was expressed as mg of Trolox equivalent (TE) per 100 g of product.

The DPPH assay was conducted following Sánchez-Moreno et al. [53], which is based on DPPH (2,2-diphenyl-1-picrylhydrazyl) solution absorbance decrease at λ = 515 nm in the antioxidant presence. The equation of the standard curve of y = 92.088x − 7.038 (R^2^ = 0.9941) was used to calculate the percentage of DPPH radical scavenging percentage and expressed as mg TE per 100 g of product.

The ability to scavenge the peroxyl radicals using fluorescein was conducted through ORAC_FL_ assay (oxygen radical absorbance capacity), which was described in a previous study [54]. Fluorometric readings were conducted using F-2700 fluorescence spectrophotometer (Hitachi, Japan) at a given excitation (493 nm) and emission wavelength (515 nm). Data was then evaluated according to a standard curve of y = 0.1728x + 0.0364 (R^2^ = 0.9995) and presented as mg TE per 100 g of product.

The PCL assay (photochemiluminescence) was performed with the use of Photochem apparatus (Analytik Jena, Germany) following the method of Gramza-Michałowska et al. [55]. The PCL assay is based on the photo-induced autooxidation inhibition of luminol, the chemical that exhibits the blue-glowing chemiluminescence, by antioxidants, mediated from the radical anion superoxide (O2^•−^) [56]. PCL evaluations were presented as antioxidant activity in lipid-soluble (ACL), water-soluble (ACW) fractions, and integral antioxidant capacity (IAC) using kits provided by the manufacturer (Analytik Jena, Germany). The result was presented as mg TE per 100 g of product.

### 2.4. Sensory Analysis

The sensory analysis of fresh gingerbread samples, with and without eggshell powder, with controlled green tea concentration (Table 1) was conducted by 15 trained panelists after the production of the samples (not more than 24 h). The sensory analysis was divided into two sections: sensory profiling and hedonic test. In sensory profiling, the panelists evaluated the appearance, aroma, texture, and taste of the fresh samples, with a range of 0 (absent) to 9 (very high/very intensive), following Rajagukguk et al. [57]. The descriptions included: appearance (surface color uniformity, glossiness, smoothness, brightness), aroma (spiciness, chocolate, green tea, sweetness, burnt, rancidity), texture (softness, humidity, porosity, crispness, adhesiveness, grittiness), and taste (sweetness, saltiness, spiciness, burnt, rancidity, strong). While in hedonic evaluation, the panelists evaluated the appearance, aroma, texture, taste, and overall, with the scale ranging from 1 (dislike extremely) to 9 (like extremely) [58]. 

Additionally, for storage analysis, the hedonic evaluation of the gingerbread samples was also conducted by 14 consumer panelists. During 2 months of storage, the gingerbread samples were kept in the dark condition under a controlled temperature (20 °C). The condition and period of storage were selected according to the approximate time that the consumer could store the product on the shelf without quality loss. The sensory analysis was conducted in the sensory analysis room, and all the consumer panelists were trained for the testing procedure. The mean, variance, and standard deviation of results were calculated for each attribute of each sample and session separately.

### 2.5. Statistical Analysis

The experiments were conducted in triplicate. One-way ANOVA and *T*-test were used, and to determine the differences between means of samples, Tukey’s multiple range test was used (*p* < 0.05). Pearson’s correlation coefficient was determined between the total phenolic content of each gingerbread treatment and the antioxidative activities. SPSS 20.0 (SPSS for Windows, SPSS Inc., Chicago, IL, USA) was applied for data analysis.

## 3. Results and Discussion

In this research, the fresh gingerbread samples (0% and 3% ESP) were compared based on basic composition and sensory analysis (appearance, aroma, texture, taste, and hedonic). Furthermore, the antioxidative activity of samples was observed during 2 months of storage and compared between treatments.

### 3.1. Basic Composition Analysis

The basic composition of gingerbread samples with and without eggshell powder is presented in Table 2. The energy value for both gingerbread samples ranged from 391.10 (gingerbread control) to 391.43 kcal/100 g (gingerbread with 3% ESP). The energy values were similar to a previous study about gluten-free gingerbread [59] and the commercial Polish gingerbread, Katarzynki (Poland). However, it is worth noticing that, in this study, the green tea powder, eggshell powder, and oat bran were added in order to increase the content of antioxidants, calcium, and fiber of gingerbread to make it more functional than commercial gingerbread. The addition of 3% eggshell powder did not affect the protein and lipid content significantly (*p* > 0.05). Alsuhaibani [60] also found a similar fat content in bread fortified with 2% eggshell powder compared to the control. In biscuit products, supplementation of 3–9% of eggshell powder did not affect the crude protein and fat content significantly [5]. Furthermore, the moisture content of gingerbread decreased after 3% of eggshell powder was incorporated. A similar result was found in Chilek et al. [61] as fortification of 6% eggshell powder decreased the moisture of white bread. Wheat flour fortified with 3% of CaCO_3_ from chicken eggshell showed lower moisture content compared to the control [62]. Therefore, the addition of 3% eggshell powder could help increase the shelf life of gingerbread by reducing the moisture content. A significant increase was also found in ash content after eggshell powder addition, which means that the mineral content was increased [61], which was in agreement with the significant increase of calcium content (*p* < 0.05) (Table 2). A similar increasing ash and calcium content trend was also found in biscuits [5], wheat bread [60], and muffin [63] fortified with eggshell powder. Therefore, the addition of chicken eggshell calcium into gingerbread could be an alternative to improve the calcium intake of the consumer. The U.S. Food and Drug Administration [64] recently updated the daily value of calcium to 1300 mg/d. A hundred grams of gingerbread with 3% ESP could fulfill around 26% of the daily value (assuming that the calcium is 46% bioavailable [4]). 

The valorization of eggshell waste as calcium supplementation in food products has been conducted by many studies, including the present study. Thanks to its affordability and abundant availability, the application of eggshell waste in food products could be carried out on household scale [4] and industrial scale [14] food industry, especially to increase people’s calcium intake. The critical point in the food industry might be the eggshell cleaning and sterilization process to guarantee that there will be no contamination of *Salmonella* or other pathogens and contaminants [16]. The substitution of calcium sources from artificial supplements to natural sources might reduce the negative effect on consumers’ bodies, and it is also cheaper than the calcium supplement. Moreover, the producers of eggshell fortified food products need to ensure the grinding process so that the sand-like texture or undesirable flavor of the product would not be perceived by consumers [18]. With the proper preparation and practices in the food industry, this could be an effective and environmentally sustainable way to reduce eggshell waste as further forecasts predict an increase (2–3%) of annual global production of eggs [16].

The dietary fiber and its fractions contents of gingerbread samples are shown in Table 3. In comparison to the control, the gingerbread sample with 3% eggshell powder possessed twice the TDF content (*p* < 0.05). A similar result was found in a study that reported a significant increase (*p* < 0.05) of fiber content in bread fortified with 2% eggshell powder [60]. The TDF consists of SDF and IDF. In accordance with TDF, both SDF and IDF contents were also higher in gingerbread with 3% eggshell powder compared to the control (*p* < 0.05). The fortification of 4% eggshell powder also increased the crude fiber content in white bread compared to the control [61]. Furthermore, the SDF (3.79% for control, 7.24% for 3% ESP) was more dominant compared to IDF (1.33% for control, 3.27% for 3% ESP). The soluble dietary fiber is linked to lowering cholesterol and blood glucose level [65]. It could also act as a prebiotic as it becomes a substrate for gut microbiota, thus it could decrease the pH in the colon during fermentation, and therefore could increase the micromineral absorption [66]. Calcium is easily absorbed in lower pH conditions [18]. While the insoluble dietary fiber may be fermented only to a limited extent in the colon, and it contributes more in bulking action and activates intestinal peristalsis [47,67].

Another concern which might affect the measurement and the increase of SDF and IDF after the eggshell powder addition to gingerbread is the mineral-binding by fibers. A study reported that lignin and cellulose, which are insoluble fibers, could bind the calcium, where the calcium binding capacity of lignin was higher than that of cellulose [68]. As previously explained, calcium is the major mineral found in eggshell powder, hence its presence in gingerbread might lead to binding process by fibers. Besides lignin and cellulose, other fibers such as neutral detergent fiber (NDF), high methoxy pectin, and guar gum (mainly composed of soluble fiber) were reported to have the ability to bind iron [69]. Other than calcium, chicken eggshell also contains other minerals, such as iron, sodium, potassium, magnesium, phosphor, zinc, and copper [70].

The NDF content, which consists of acid detergent fiber (ADF, including lignin and cellulose) and hemicellulose, was also determined. Both NDF and hemicellulose content of gingerbread with 3% eggshell powder were higher than the control (*p* < 0.05). To date, the research about fiber fraction content of chicken eggshells, especially using Van Soest assay, has not been reported yet. However, Al-awwal and Ali [70] and Ajala et al. [71] reported that the crude fiber content of chicken eggshells was 3% and 4.38%, respectively. Cellulose and lignin are included in crude fiber. Although chicken eggshell has 3–4.38% of crude fiber, an insignificant increase of lignin and cellulose content was found in gingerbread with 3% eggshell powder compared to the control (*p* > 0.05).

### 3.2. Total Phenolic Content and Antioxidative Activity Analysis

The results of total phenolic content (TPC) and antioxidative activity (ABTS, DPPH, ORAC_FL_, PCL assays) analysis are shown in Table 4. In general, the longer the storage time, the lower the antioxidative activity, as well as total phenolic content. During 2 months of storage, a significant decrease of TPC was found in the gingerbread control (from 215.59 to 198.47 mg GAE/100 g) and 3% ESP (from 214.01 to 196.74 mg GAE/100 g) (*p* < 0.05). The ABTS radical cation scavenging activity was decreased significantly (*p* < 0.05), about 12%, after 1 month of storage in both samples. However, no significant difference in scavenging activity was found between those after 1 month and 2 months of storage. In DPPH radical scavenging activity assay, the scavenging activity decreased significantly (*p* < 0.05) around 4–5% every 1 month of storage, in both the control and 3% ESP samples. ORAC_FL_ assay, which is widely used to determine the antioxidant capacity written on food labels, showed similar results as a significant decrease of antioxidant was seen after 1 month (for the control sample) and after 2 months of storage (for the 3% ESP sample) (*p* < 0.05). The ORAC_FL_ values ranged from 996.73 to 1224.43 mg TE/100 g for the control and 1046.38 to 1194.88 mg TE/100 g for gingerbread with 3% ESP. All of those results were in accordance with the result of total phenolic content, which exhibited lower total phenolic content along with longer storage time. During storage, the available antioxidant in the samples scavenged the free radicals, which are responsible for various oxidation reactions by, for example, donating the proton or electron from the hydroxyl groups of phenolics [72]. Due to the reduction of free radicals, the scavenging activity is also reduced after a longer storage time [73]. A similar trend was found in cookies fortified with 5.5% ground green tea leaves during 3 months of storage [19], especially in ABTS and DPPH assays. However, the authors reported that total phenolic content and antioxidative activity using ORAC_FL_ did not significantly decrease during 3 months of storage.

In comparison between gingerbread control and that fortified with 3% eggshell powder, the addition of 3% eggshell powder to the gingerbread samples did not significantly influence the total phenolic content, which was in agreement with antioxidative activity, particularly using ABTS and ORAC_FL_ assays. However, the total phenolic content was not in agreement with an antioxidative activity using the DPPH assay. Different from ABTS and ORAC_FL_ assay, in the DPPH assay, there were 4–5% significant decreases of antioxidative activity after the eggshell was added in the same storage time. Despite several studies reporting that eggshell and eggshell membrane peptides could increase the antioxidant activity using DPPH assay [74,75,76], a study also showed lower DPPH radical scavenging activity of fresh-cut muskmelon dipped in a solution of 2% calcium chloride extracted from eggshell and commercial calcium chloride, compared to the control without treatment [73].

The determination of antioxidative activity using different in vitro assays shows different results. The antioxidant compounds respond differently to different radical or oxidant compounds [77], in this case, ABTS cation radical and DPPH radical. ABTS assay is based on electron transfer, and hydrogen atom transfer may also apply, while the DPPH method is based on electron transfer [52]. When the antioxidant activity takes place, the ABTS assay monitors the decolorization and the decrease of absorbance at 743 nm, while the DPPH assay monitors the color-changing (purple to yellow or colorless) and the decrease of absorbance at 515–528 nm [78]. A study reported different IC50 values of various antioxidants between ABTS and DPPH assay in comparison with the HPLC method [79]. The authors concluded that ABTS and DPPH assays are sufficient for measuring antioxidative activity when the spectrum of examined antioxidant or real biological system does not coincide with the wavelength used to monitor radical concentration changes. Otherwise, the difference between true and spectrophotometrically estimated IC50 values depends not only on its extinction coefficient of the monitored wavelength but also on its residue concentration in the measuring system. For example, anthocyanins have strong absorption at 500–550 nm, which is similar to the wavelength for DPPH assay, thus, they could interfere with the results and the interpretation of antioxidative activity [52]. Furthermore, a study explained that each radical shows different affinities although in the same sample. ABTS has a higher affinity with hydrophilic, lipophilic compounds, and hydrogen atom donors, while DPPH has a higher affinity for lipophilic compounds and less affinity for compounds containing aromatic rings with only -OH groups [80]. Floegel et al. [81] also compared the antioxidative activity of various food products measured by ABTS and DPPH assays to the USDA ORAC database and reported that the ABTS assay reflects the antioxidant activity in various food products (particularly plant-based foods) better than DPPH assay. The authors reported that the result from the ABTS assay has a stronger correlation with the ORAC USDA database.

The photochemiluminescence assays (PCL) are rapid, simple, and reproducible, which is useful as the biomonitoring tool, particularly in the food technology and nutrition field [56]. In PCL assay, the antioxidative capacity of water-soluble fraction (ACW) and lipid-soluble fraction (ACL) were determined. Generally, the hydrophilic antioxidants react with oxidants in the cell cytoplasm and blood plasma, while hydrophobic antioxidants protect the cell membranes from lipid peroxidation [82]. The result (Table 4) showed that the ACL in gingerbread with 3% ESP was significantly higher during 2 months of storage in comparison to the control. Furthermore, the ACW in the fresh control sample was higher than the ACW in fresh gingerbread with 3% ESP, but there was no significant difference of activity between them after 1 and 2 months of storage. During 2 months of storage, regardless of the significancy, the ACL of both samples showed an increasing trend; while in contrast, ACW decreased. The increase of ACL was also found in cookies fortified with ground green and yellow tea and control cookies after 1 month of storage; while after 3 months, the ACL showed significant and insignificant decreasing trend, depending on the type of tea [19]. The pressed cake, the by-product of flaxseed oil extraction, exhibited increased α-tocopherol (a lipid soluble antioxidant) content after 3 months of storage, which might be due to the conversion from γ-tocopherol to α-tocopherol during storage [83]. Afterwards, the authors found that in the sixth month, the α-tocopherol content of the cake showed decreasing trend. In comparison to γ-tocopherol, α-tocopherol is generally known as a more potent antioxidant in lipid peroxidation inhibition [84], which might then affect the ACL. The oat bran, as one of the ingredients of gingerbread samples in the present study, was reported to contain oil containing both α and γ -tocopherol [85]. This might be the reason why the ACL of both gingerbread samples increased during 2 months of storage. However, additionally, the ACL might decrease if longer storage time (3 months or more) was observed, which was reported by a previous study [19].

The integral antioxidative capacity (IAC) was also determined as it represents the sum of antioxidative activity of water-soluble and lipid-soluble antioxidants. Generally, there was no significant difference of IAC between the control and gingerbread with 3% ESP, especially for the fresh ones and after 1 month of storage. However, after 2 months of storage, the result showed higher IAC in gingerbread with 3% ESP compared to the control. Comparing between storage times, the IAC in the control sample decreased significantly after 1 month of storage (561.90 to 481.14 mg TE/100 g), then no significant difference was found after 2 months of storage. While for gingerbread with 3% ESP, no significant changes were noticed during 2 months of storage time. To summarize, in general, gingerbread with 3% ESP could be a potential and promising functional food with good antioxidative activity and improved calcium content. Additionally, prior studies [19,55] reported that green, yellow, red, black, and white tea leaves, as well as tea leaves addition to cookies, increased the IAC, ACW, and ACL of the products. Thus, green tea addition in both the gingerbread control and gingerbread with 3% ESP might also contribute to the improvement of the antioxidant activity, especially in PCL assay.

The correlation between the TPC and the antioxidative activities of gingerbread samples was also checked statistically using Pearson’s correlation coefficient (Table 5). In both gingerbread samples, the TPC was strongly correlated with the antioxidative activities using ABTS, DPPH, ORAC_FL_, PCL-ACW, and PCL-IAC. However, the TPC of both gingerbread samples showed a negative correlation with the antioxidative activity using PCL-ACL assay. The Folin-Ciocalteu method was used for the determination of TPC. It was reported that the Folin–Ciocalteu chromophore, which is multivalent-charged phosphor-tungsto-molybdate, has a higher affinity to water and hydrophilic compounds than the lipophilic compounds, which make it more capable of measuring the hydrophilic antioxidants [86]. Therefore, the assay is unable to determine the total phenolics of lipophilic antioxidants. On the other hand, PCL-ACL determines the antioxidative activity of lipophilic antioxidants. This might be the reason why TPC has a negative correlation with PCL-ACL, and in contrast, a positive correlation was found between TPC and PCL-ACW. The result was not in accordance with Abdullakasim et al. [87] who found positive but rather weak correlations between TPC (using Folin–Ciocalteu method) and ACW and ACL of various Thai beverages, with r-values of 0.417 and 0.459, respectively. The different samples might affect the different results due to different compositions. It is worth noticing that the chemical characteristic of the Folin–Ciocalteu reagent is not specific to phenolic compounds but can be also interfered by other compounds such as sugars, ascorbic acid, aromatic amines, sulfur dioxide, organic acids, Cu(I), Fe(II), etc. [87,88]. Consumers also need to consume functional food products with high antioxidative activity, not only high in calcium content. Food products with high antioxidative activity may contribute to the reduction of oxidative stress. It is worth noticing that oxidative stress leads to osteoporosis. In this research, gingerbread with 3% ESP exhibited antioxidative activity, which has potential to reduce the risk of osteoporosis.

### 3.3. Sensory Analysis

#### 3.3.1. Sensory Analysis of Fresh Gingerbread Samples

**Figure 4:**
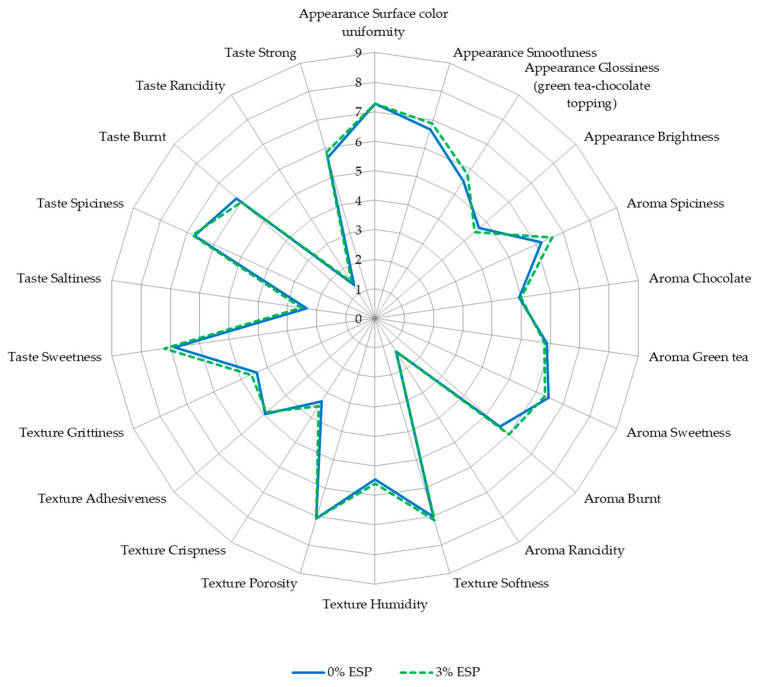
Sensory profiling of fresh gingerbread samples; ESP: egg shell powder.

The appearance, aroma, texture, and taste of fresh gingerbread samples, with and without eggshell powder, with controlled green tea concentration, are presented in Figure 4. According to the evaluators, the addition of 3% eggshell powder did not significantly affect the appearance of the gingerbread samples in all descriptors. Both samples had rather low overall brightness (4.47–4.67), which is caused by the brown color of gingerbread itself. The caramelization of sugar and Maillard reaction, which is the reaction between the free amino group of lysine, peptides, or protein and carbonyl groups of reducing sugars, might take place during the baking process of bread products, which accelerates the browning reaction [89,90]. Another study also evaluated that the incorporation of green tea powder resulted in a darker color in the donut’s crust [91].

In terms of aroma, the addition of 3% eggshell powder did not significantly affect the aroma of the gingerbread samples in all descriptors. In both samples, the two dominant aromas were spicy and sweet, followed by green tea, burnt, and chocolate aroma. While the rancid aroma of both samples was the least intensive aroma compared to other descriptors, and no significant difference of rancidity was found after the eggshell powder addition (*p* > 0.05). The ingredients of both samples, especially gingerbread spice mix and green tea powder might be strong contributors to this aroma profiling. However, it is important to note that the rancidity might be more intensive along with the longer storage time due to the oxidation process of butter-containing products [92].

In texture profiling, the addition of 3% eggshell powder did not significantly affect the texture of the gingerbread samples in all descriptors. The lowest intensive descriptor in both samples was crispness (3.33–3.53). The panelists assessed that both samples were rather highly intensive in softness and porosity descriptors. Interestingly, the grittiness of gingerbread incorporated with 3% eggshell powder was not significantly different from the control. This means that the eggshell powder addition (3% *w*/*w* of flour) could improve the calcium content without significant grittiness changes. This result was in agreement with Ray et al., who suggested the supplementation of eggshell powder up to 6% in cake products, with consideration of calcium content, texture, and sensory properties [41]. However, the panelists could still sense a rather low intensity of grittiness of both samples (4.40–4.60). This might be due to the coarse properties of oat bran [93] as one of the ingredients of both samples. A textural study reported that the insoluble fiber part of oat bran contributes to grittiness and chalky mouthfeel perception [94]. The insoluble fiber content of oat bran is 9.73%, while the soluble fiber content is 7.17% [95]. Therefore, oat bran was not only contributing as a prebiotic which could support calcium absorption but also as the ingredient to hide the grittiness from the eggshell powder. Furthermore, the insoluble fiber part of oat bran also contributes to the dry mouthfeel [94], which might result in moderate humidity of the samples (5.47–5.60).

The elderly need food products which are easy to swallow. A study reported that soft and moist food products are recommended for the elderly, while adhesive texture must be avoided as dry and adhesive food products will make it hard to propel the foods through the pharynx [96]. In this study, the gingerbread sample, especially with 3% eggshell powder, showed rather high softness (7.13), moderate humidity (5.60), and rather low adhesiveness (4.87), which is in accordance with the recommendation for the elderly. Moreover, considering the higher calcium content, gingerbread with 3% eggshell powder could be a potential functional food for the elderly to reduce the risk of osteoporosis.

The addition of 3% eggshell powder did not significantly affect the taste of the gingerbread samples in all descriptors. This result was in agreement with Brun et al., who concluded that the best way to incorporate calcium from eggshells was by adding it to bread, pizza, and spaghetti as there were minimal changes in taste and texture [4]. The result was also in accordance with the aroma profiling as the sweet and spicy taste were dominant, while the rancid taste was the least intensive taste. About 75–95% of what we taste actually comes from the sense of smell [97].

**Figure 5:**
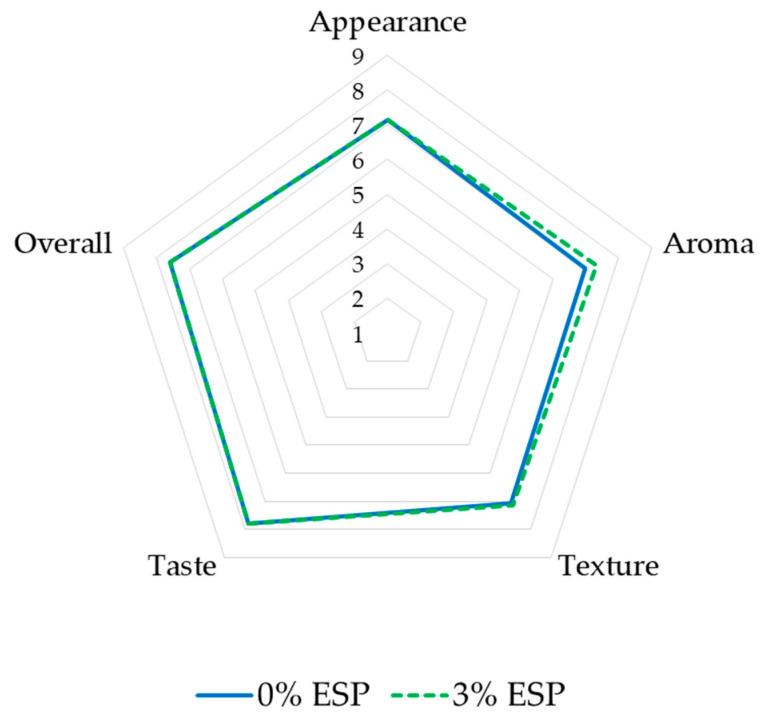
Hedonic evaluation of fresh gingerbread samples; ESP: eggshell powder.

Fresh gingerbread hedonic evaluation (Figure 5) ranged from 7.00 to 7.80, showing moderate-high consumer acceptability. The result also showed no significant difference in each aspect between the one with 3% eggshell powder addition and the control. The result was in accordance with prior studies. Supplementation of 5–15% of both white and brown chicken eggshell to bread strips showed no significant hedonic evaluation in all aspects (color, taste, aroma, texture, and overall acceptability) compared to control bread strips [98]. Furthermore, the sensory evaluation of white bread fortified with 1–1.5% [99] and 2% eggshell powder [61] also exhibited high consumer acceptance. Overall, with the positive results from sensory profiling (appearance, aroma, texture, and taste) and hedonic evaluation, the gingerbread fortified with 3% ESP is also able to be consumed and accepted by the elderly and therefore could potentially help in reducing the risk of osteoporosis.

#### 3.3.2. Hedonic Evaluation during Storage Analysis

**Figure 6:**
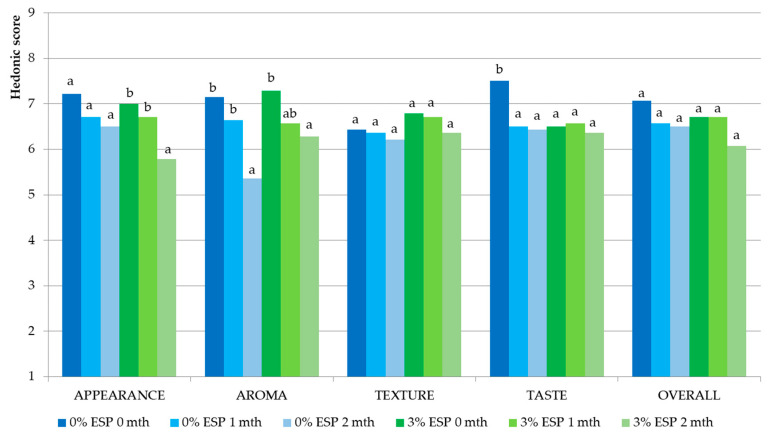
Hedonic evaluation of gingerbread samples during 2 months of storage. ESP: eggshell powder, mth: month of storage. Different lowercase letters in the same concentration of ESP and sensory descriptor indicate significant difference (*p* < 0.05).

The hedonic evaluation of appearance, aroma, texture, taste, and overall of gingerbread samples, with and without eggshell powder, during 2 months of storage is presented in Figure 6. During storage, the hedonic score showed moderate consumer acceptability as it ranged from 5.4 to 7.5. Generally, all the hedonic scores showed decreasing trends during 2 months of storage. The hedonic scores of the aroma of both treatments decreased significantly (*p* < 0.05). However, no significant decrease was observed in texture and overall scores of both treatments (*p* > 0.05). For appearance, a significant decrease of the hedonic score was observed in the second month of storage of gingerbread with 3% ESP (*p* < 0.05). In terms of taste, the hedonic score decreased around 13% (*p* < 0.05) in gingerbread without eggshell powder after 1 month of storage, compared to 0 month, while no significant decrease was found in that with 3% ESP during 2 months of storage. Comparing between 0 and 2 months of storage, the biggest decrease (25%) was found in the aroma of the control gingerbread, followed by the appearance of gingerbread fortified with 3% ESP (17%). In the preparation of gingerbread samples, butter was added. During the storage of fat-containing products, the oxidation of fat might take place, which produces the off-flavor, such as rancid flavor [100]. However, it was reported that the addition of green tea and yellow tea leaves to cookies decreased the rate of fat oxidation thanks to their antioxidative activity [19]. In terms of appearance, Khoshakhlagh et al. [101] explained that the color changes in bread products during storage might be caused by the moisture loss and the moisture transfer from crumb to crust, which then brightened the crust color. This might also then influence and decrease the hedonic score of the appearance of gingerbread samples.

## 4. Conclusions

Eggshell is a food waste which may cause environmental and health problems if it is not managed properly. In this study, the innovative functional food based on gingerbread enriched with antioxidants from green tea and chicken eggshell calcium was developed. The addition of 3% (*w*/*w* of wheat flour) eggshell powder improved the ash and calcium content of the gingerbread without significantly changing the appearance, aroma, texture, taste, and the hedonic score of gingerbread in comparison with gingerbread without eggshell powder. About 26% DV of calcium could be fulfilled by consuming 100 g of gingerbread fortified with 3% ESP (assuming that the calcium is 46% bioavailable). The addition of eggshell powder significantly increased the content of hemicellulose, soluble, insoluble, and total dietary fiber of gingerbread. It was also observed that the soluble dietary fiber was predominant in gingerbread fortified with 3% ESP, which might potentially have a role as a prebiotic agent and then might increase the bioavailability of microminerals, including calcium. The fortification of 3% ESP to the gingerbread did not change the antioxidative activity in ABTS and ORAC_FL_ assay. The antioxidative activities and hedonic scores of both gingerbread samples decreased during 2 months of storage. However, both samples still possessed antioxidative activity and moderate consumer acceptability after 2 months of storage. In industrial production, the sterilization and grinding process of eggshells must be conducted properly to obtain safe and sensorially acceptable eggshell powder. Although the gingerbread in this study contained increased calcium content and antioxidative activity, the sugar consumption should be reduced in further studies following the current health trend in reducing high sugar intake. The information observed in this research could be useful for scientific knowledge, especially as one of the alternative ways to solve eggshell waste problem by incorporating it into gingerbread to decrease calcium deficiency and the risk of osteoporosis, which are global health problems. 

## Figures and Tables

**Table 1:** Formulation of gingerbread.

Ingredients	Weight (g)	
	Gingerbread	
	0% ESP	3% ESP
Mixture 1		
Milk 3.2% fat	45.00	45.00
Unrefined cane sugar	5.00	5.00
Dry yeast	2.00	2.00
Mixture 2		
Wheat flour type 650	140.00	140.00
Oat bran	35.00	35.00
Unrefined cane sugar	30.00	30.00
Salt	2.00	2.00
Honey	21.00	21.00
Egg	50.00	50.00
Baking soda	2.00	2.00
Gingerbread spice mix	20.00	20.00
Butter 82% fat	19.00	19.00
Eggshell powder *	0.00	4.20
Mixture 3 (green tea-chocolate topping)		
White chocolate	68.00	68.00
Green tea powder **	3.40	3.40

Notes: 25 pcs gingerbread per treatment after baking and topping addition (±16.5 g/pcs). ESP: eggshell powder; pcs: pieces; *—3% (*w*/*w*) of wheat flour; **—4% (*w*/*w*) of white chocolate.

**Table 2:** Basic composition of gingerbread samples.

Nutrient (%)	Sample	
	0% ESP	3% ESP
Protein	8.67 ± 0.07 a	8.76 ± 0.08 a
Lipid	12.19 ± 0.05 a	12.30 ± 0.09 a
Carbohydrate	61.76 ± 0.10 b	61.34 ± 0.11 a
Ash	1.46 ± 0.01 a	3.40 ± 0.05 b
Moisture	15.92 ± 0.11 b	14.19 ± 0.02 a
Energy value (kcal/100 g)	391.43	391.10
Mineral (mg/100 g)		
Calcium	86.71 ± 1.00 a	729.42 ± 31.95 b

ESP: eggshell powder. Different lowercase letters in the same row indicate significant difference (*p* < 0.05).

**Table 3:** Dietary fiber and its fractions contents in fresh gingerbread samples.

Dietary Fiber (%)	Sample	
	0% ESP	3% ESP
NDF	4.73 ± 0.30 a	7.09 ± 0.62 b
ADF	2.74 ± 0.20 a	3.41 ± 0.41 a
ADL	1.19 ± 0.24 a	1.29 ± 0.26 a
ADC	1.55 ± 0.44 a	2.12 ± 0.21 a
Hemicellulose	1.99 ± 0.44 a	3.68 ± 0.89 b
SDF	3.73 ± 0.14 a	7.13 ± 1.30 b
IDF	1.33 ± 0.25 a	3.27 ± 0.15 b
TDF	5.06 ± 0.24 a	10.41 ± 1.43 b

ESP: eggshell powder, NDF: neutral dietary fiber, ADF: acidic dietary fiber, ADL: acid detergent lignin, ADC: acid detergent cellulose, SDF: soluble dietary fiber, IDF: insoluble dietary fiber, TDF: total dietary fiber. Different lowercase letters in the same row indicate significant difference (*p* < 0.05).

**Table 4:** Total phenolics content and antioxidative activity of fresh and stored gingerbread samples.

Assay			Storage Time (month)	0% ESP	3% ESP
TPC		(mg GAE/100 g)	0	215.59 ± 2.69 cA	214.01 ± 1.18 cA
			1	204.75 ± 2.51 bA	204.67 ± 0.99 bA
			2	198.47 ± 2.06 aA	196.74 ± 0.58 aA
ABTS		(mg TE/100 g)	0	453.79 ± 1.18 bA	448.82 ± 5.11 bA
			1	402.73 ± 1.19 aA	397.40 ± 6.23 aA
			2	396.40 ± 13.66 aA	388.83 ± 12.48 aA
DPPH			0	388.13 ± 5.06 cB	370.44 ± 1.78 cA
			1	372.13 ± 1.23 bB	354.94 ± 6.43 bA
			2	354.93 ± 5.69 aB	340.98 ± 3.38 aA
ORAC_FL_			0	1224.43 ± 39.63 bA	1194.88 ± 66.1 bA
			1	1003.80 ± 20.36 aA	1062.21 ± 21.67 abA
			2	996.73 ± 28.43 aA	1046.38 ± 31.6 aA
	ACW		0	305.49 ± 18.19 bB	244.99 ± 0.54 bA
			1	219.95 ± 11.25 aA	222.68 ± 0.36 aA
			2	216.34 ± 1.12 aA	214.36 ± 4.39 aA
PCL	ACL		0	256.41 ± 6.63 aA	289.40 ± 4.35 aB
			1	261.19 ± 9.31 aA	294.61 ± 8.30 aB
			2	287.86 ± 2.82 bA	302.70 ± 2.86 aB
	IAC		0	561.90 ± 16.15 bA	536.38 ± 4.30 aA
			1	481.14 ± 14.69 aA	517.59 ± 12.07 aA
			2	505.56 ± 2.86 aA	516.89 ± 0.37 aB

ESP: eggshell powder, TPC: total phenolics content, ABTS: 2,2′-azinobis(3-ethylbenzothiazoline-6-sulfonic acid) cation assay, DPPH: 2,2-diphenyl-1-picrylhydrazyl assay, ORAC_FL_: oxygen radical absorbance capacity assay, PCL: photochemiluminescence assay, ACW: water-soluble antioxidative capacity, ACL: lipid-soluble antioxidative capacity, IAC: integral antioxidative capacity, GAE: gallic acid equivalent, TE: Trolox equivalent. Different lowercase letters in the same column indicate significant difference in each analyzed method (*p* < 0.05). Different uppercase letters in the same row indicate significant difference in each analyzed method (*p* < 0.05).

**Table 5:** Pearson’s correlation coefficient between total phenolics content and antioxidative activities of gingerbread samples.

Samples	TPC with	Pearson’s r	Sig. (2-Tailed)
0% ESP	ABTS	0.870	*p* < 0.01
	DPPH	0.894	*p* < 0.01
	ORAC_FL_	0.923	*p* < 0.01
	PCL-ACW	0.888	*p* < 0.01
	PCL-ACL	−0.816	*p* < 0.01
	PCL-IAC	0.707	*p* < 0.05
3% ESP	ABTS	0.901	*p* < 0.01
	DPPH	0.958	*p* < 0.01
	ORAC_FL_	0.820	*p* < 0.01
	PCL-ACW	0.963	*p* < 0.01
	PCL-ACL	−0.726	*p* < 0.05
	PCL-IAC	0.814	*p* < 0.05

ESP: eggshell powder, TPC: total phenolics content, ABTS: 2,2′-azinobis(3-ethylbenzothiazoline-6-sulfonic acid) cation assay, DPPH: 2,2-diphenyl-1-picrylhydrazyl assay, ORAC_FL_: oxygen radical absorbance capacity assay, PCL: photochemiluminescence assay, ACW: water-soluble antioxidative capacity, ACL: lipid-soluble antioxidative capacity, IAC: integral antioxidative capacity.

## Data Availability

The data used to support the findings of this study can be made available by the corresponding author upon request.

## Abbreviations

ABTS: 2,2′-azinobis(3-ethylbenzothiazoline-6-sulfonic acid) cation assay, ACW: water-soluble antioxidative capacity, ACL: lipid-soluble antioxidative capacity, ADC: acid detergent cellulose, ADF: acidic dietary fiber, ADL: acid detergent lignin, CaCO_3_: calcium carbonate, DPPH: 2,2-diphenyl-1-picrylhydrazyl assay, ESP: eggshell powder, GAE: gallic acid equivalent, IAC: integral antioxidative capacity, IDF: insoluble dietary fiber, mth: month of storage, NDF: neutral dietary fiber, ORAC_FL_: oxygen radical absorbance capacity assay, PCL: photochemiluminescence assay, pcs: pieces, SDF: soluble dietary fiber, TDF: total dietary fiber, TE: Trolox equivalent, TPC: total phenolics content, UHT: ultra-high temperature.
